# Editorial: Biomedical applications and health impacts of emerging nanostructured materials

**DOI:** 10.3389/fbioe.2023.1282946

**Published:** 2023-09-01

**Authors:** Jianbo Jia, Qingxin Mu, Hongyu Zhou

**Affiliations:** ^1^ Key Laboratory for Water Quality and Conservation of the Pearl River Delta, Ministry of Education, Institute of Environmental Research at Greater Bay, Guangzhou University, Guangzhou, China; ^2^ Department of Pharmaceutics, University of Washington, Seattle, WA, United States

**Keywords:** nanomedicine, nanotoxicology, emerging nanostructures, nano-cell interaction, biological effect

Innovative nanostructures have been engineered for various biomedical applications, such as the diagnosis and therapy of diseases and the remediation of hazardous medical waste, presenting a transformative avenue for challenges in traditional biomedicine. While such bio-responsive exotic nanostructures may also have negative health impacts, it becomes crucial to simultaneously advance their biomedical applications while thoroughly investigate the health implications. With a specific focus on the latest advances in emerging nanostructures for biomedical applications and their health concerns, the Research Topic “*Biomedical applications and health impacts of emerging nanostructured materials*” records five articles including two Original Research articles, one Review, one Mini Review, and one Opinion.

The two Original Research articles highlight the medical applications of emerging metal-organic framework (MOF)-based nanostructures. Specifically, Yang et al. constructed an miR-200c-3p@ZIF-8 nanostructure in one step using a Y-shaped microfluidic chip for effective osteoarthritis treatment. At the cellular level, the authors verified that the ZIF-8 vectors had low cytotoxicity and high miRNA loading efficiency, and improved the cellular uptake and endosomal escape of miR-200c-3p, an osteoarthritis therapeutic microRNA. More importantly, the enhanced delivery and stable expression of miR-200c-3p suppressed the protein expression of various inflammatory cytokines in LPS-induced chondrocytes, confirming the promising application of this emerging nanostructure in the treatment of osteoarthritis. Taking ciprofloxacin as a typical medical waste, Geng et al. compared the degradation efficiencies of medical waste by four types of Fe-based MOFs with different structures to identify the key factors affecting the stability of the MOF structures during the peroxodisulfate activation processes. The authors reported that active species (i.e., OH· and SO_4_^−^·), oxidant reagent, and low pH negatively affected the stability of MOF structures, and the Fe(II)-MOFs exhibited the best stability performance among the tested nanostructures. This work provides a new insight into the future development of Fe-based MOFs for the remediation of hazardous medical waste.

Regarding the health concerns of the emerging nanostructures, Xuan et al. provided an updated overview of the key factors that modulate the biological effects of nanoparticles. In their review, the influences of not only the primary characterizations of particle size, shape, chemical composition, and surface modification, but also the secondary modifications with proteins on the biological effects of nanoparticles, were discussed. The most recent advances in the underlying mechanisms involved in nano-bio interactions were also summarized. In another Mini Review, Zhou et al. discussed the potential health risks of engineered nanoparticles, with a particular focus on their interference with pulmonary inflammation. According to the authors, certain nanoparticles themselves are likely to be inflammation inducers, causing or exacerbating the inflammatory responses in the lung. However, through careful engineering, they could also serve as vectors of anti-inflammatory drugs, posing therapeutic potentials for lung inflammation.

In addition, Zhang et al. contributed an Opinion article to this Research Topic, prospecting the potential application of nanotechnology in advanced cryopreservation of biological samples. In brief, the nanoparticle-assisted delivery methods have helped overcome the major limitation of the ultra-low permeability of impermeable cryoprotectants, and methods like nanowarming could help achieve rapid and uniform rewarming while avoiding the adverse effects of devitrification on biological samples. In cooperation with the novel automatic washing techniques, efficient and high-quality cryopreservation methods are expected to be realized in the near future.

Although this Research Topic encompasses several cutting-edge breakthroughs, the prevailing challenges persist. Developing innovative nanostructures to effectively address major challenges remains a top research strategy with significant implications for advanced biomedical applications. Besides, the need to tackle essential concerns related to the bioactivity, compatibility, and nano-bio interfacial properties of emerging nanostructures remains a prominent area that requires comprehensive investigation and resolution.

